# Practical Departments

**Published:** 1904-08-06

**Authors:** 


### PRACTICAL DEPARTMENTS.

### COST OF CASUALTY CASES IN PROVINCIAL GENERAL HOSPITALS.

"HOSPITAL SECRETARY" writes:—I am directed by the chairman to ask you to kindly inform me what the cost of each casualty is considered to be in provincial general hospitals.

[The cost of each casualty in Provincial General Hospitals has never been ascertained so far as we are aware. We do not see why it should differ from the cost of a casualty patient in other general hospitals, and "Hospital Secretary" must be aware that it is impossible, with any regard to accuracy, to lump all casualty cases together and so arrive at the cost of each. The cost must necessarily depend upon the character of each case, and that can only be ascertained by a careful analysis of the books for a given period by the authorities of each hospital.—ED. THE HOSPITAL.]

